# Low Energy Intake Diagnosed Using the Harris–Benedict Equation Is Associated with Poor Prognosis in Elderly Heart Failure Patients

**DOI:** 10.3390/jcm12227191

**Published:** 2023-11-20

**Authors:** Akira Taruya, Tsuyoshi Nishiguchi, Shingo Ota, Motoki Taniguchi, Manabu Kashiwagi, Yasutsugu Shiono, Ke Wan, Yasushi Ino, Atsushi Tanaka

**Affiliations:** 1 Department of Cardiovascular Medicine, Wakayama Medical University, Wakayama 641-0012, Japan; 2 Department of Cardiovascular Medicine, Shingu Municipal Medical Center, Shingu 647-0072, Japan; 3 Department of Internal Medicine, Wakaura Central Hospital, Wakayama 641-0054, Japan; 4 Clinical Research Support Center, Wakayama Medical University Hospital, Wakayama 641-0012, Japan

**Keywords:** elderly patients, heart failure, energy intake, Harris–Benedict equation, prognosis

## Abstract

Introduction: Insufficient nutrient intake is a strong independent predictor of mortality in elderly patients with heart failure. However, it is unclear to what extent energy intake affects their prognosis. This study investigated the association between patient outcomes and actual measured energy intake in elderly patients (≥65 years) with heart failure. Methods: This study enrolled 139 elderly patients who were hospitalized with worsening heart failure at Shingu Municipal Medical Center, Shingu, Japan, between May 2017 and April 2018. Energy intake was evaluated for three days (from three days prior to the day of discharge until the day of discharge). Based on basal energy expenditure calculated using the Harris–Benedict equation, the patients were classified into a low-energy group (*n* = 38) and a high-energy group (*n* = 101). We assessed the prognosis in terms of both all-cause mortality and readmission due to worsening heart failure as a primary outcome. Results: Compared to the patients in the high-energy group, the patients in the low-energy group were predominantly female, less frequently had smoking habits and ischemic heart diseases, and had a higher left ventricular ejection fraction. The low-energy group had higher mortality than the high-energy group (*p* = 0.028), although the two groups showed equivalent event rates of the primary outcome (*p* = 0.569). Conclusion: Calculations based on the Harris–Benedict equation revealed no significant difference in the primary outcome between the two groups, with a secondary outcome that showed worse mortality in the low-energy group. Given this result, energy requirement-based assessments using the Harris–Benedict equation might help in the management of elderly heart failure patients in terms of improved life outcomes.

## 1. Introduction

Insufficient nutrient intake is a strong independent predictor of mortality in patients with heart failure (HF) [1,2]. The Meta-Analysis Global Group in Chronic Heart Failure (MAGGIC) risk score [3] is a widely used tool for predicting mortality after the discharge of patients with HF [4,5]. It is calculated using a number of individual risk factors, such as serum sodium, sex, and preserved or reduced left ventricular ejection fraction (LVEF). However, nutritional factors are not included in the MAGGIC risk score. Meanwhile, although there are a number of malnutrition scores, including the Geriatric Nutritional Risk Index (GNRI) [6] and the Controlling Nutritional Status (CONUT) Index [7], the typical presentation of malnutrition is a loss of appetite, which represents an independent factor of hospital mortality [8]. Energy deficits are associated with an increased proportion of infections in patients in intensive care units [9]. However, it is unclear to what extent energy intake affects the prognosis of HF.

The Harris–Benedict equation (HBE) [10] is considered to be the best equation to predict basal energy expenditure (BEE) [11]; however, in elderly patients (≥65 years), it has been reported to underestimate energy requirements [12]. Conversely, the HBE has been shown to overestimate BEE in patients with HF [13]. At present, the best indicators of the appropriate energy needs of elderly patients with HF remain unclear.

We hypothesized that the HBE would be reliable for elderly patients with HF and that elderly patients with inadequate energy intake with HF would have a much worse prognosis than those with adequate energy intake. This study investigated the association between the outcomes and actual measured energy intake of elderly patients with HF.

## 2. Materials and Methods

### 2.1. Subjects

**Figure 1:**
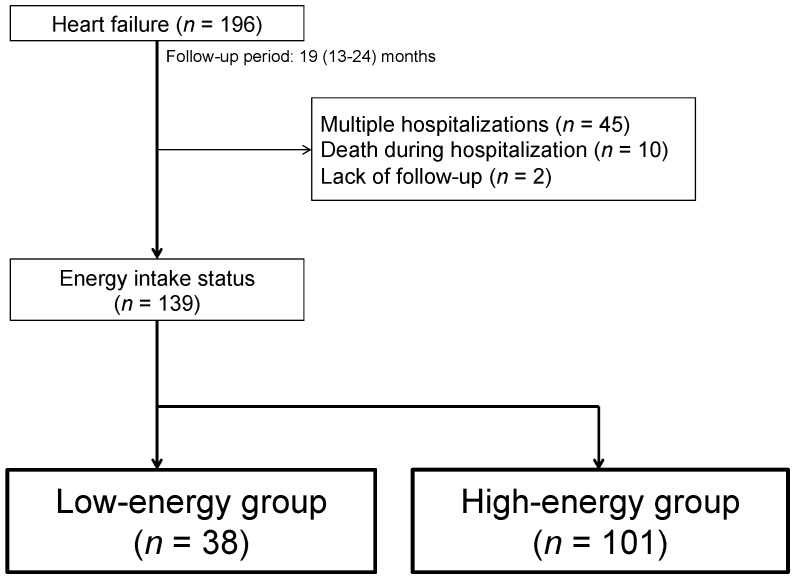
Flowchart of the study population and design.

We included 196 consecutive elderly HF patients (aged 65 years or older) without severe valvular disease, congenital disease, complete atrioventricular block, pericardial disease, primary pulmonary hypertension, or acute coronary syndrome who were admitted to Shingu Municipal Medical Center, Shingu, Japan, due to worsening HF between May 2017 and April 2018 and discharged home. HF was defined as follows: HF symptoms according to the Framingham criteria and increased plasma concentration of brain natriuretic peptide (BNP) (>100 pg/mL) at admission. We excluded patients who died during index hospitalization (*n* = 10), patients already enrolled in the study who were admitted to the hospital with an HF exacerbation during the follow-up period (1 year) (to avoid double counting) (*n* = 45), and those who could not be followed up (*n* = 2). Finally, 139 patients were enrolled in the present study (Figure 1).

This study was approved by the Shingu Municipal Medical Center Ethics Committee (Number: 65) and was conducted in accordance with the Declaration of Helsinki. Informed consent was obtained from all patients in accordance with the committee guidelines.

### 2.2. Patient Characteristics

Clinical and laboratory data were retrospectively collected from the patients’ records. BNP was measured with a commercially available kit (BNP-JP 471680R03, Abbott Japan, Tokyo, Japan). The LVEF was quantitated via echocardiogram at admission. Medical histories and findings in the physical examination were also collected from the records. Ischemic heart disease was defined as any medical history of acute coronary syndrome, percutaneous coronary intervention, or coronary artery bypass surgery or a diagnosis of myocardial ischemia based on invasive/noninvasive diagnostic tests.

### 2.3. Dietary Assessment

Meals with a set total energy content were provided, and their intake was directly observed. Nurses in our cardiovascular section visually estimated dietary intake after each meal (main dishes and staple meals were estimated separately). This is standard practice in most hospitals in Japan [14], and nurses are trained in standardized visual dietary intake assessment based on protocols implemented throughout the Shingu Municipal Medical Center. The consumption of each dietary item is recorded as a percentage (expressed in ×10%) of the total amount. From the data recorded in these medical charts, the overall average percentage intake of main and side dishes was calculated for each meal. With reference to a previous study [15], the average energy intake was estimated and calculated under the supervision of a dietitian from the average percentage intake of the three meals (breakfast, lunch, and dinner) obtained from the medical record review and the total energy of the meals provided during the three days before discharge. BEE was calculated using the following HBE formulas [10]: 66.5 + 13.76 × weight (kg) + 5.003 × height (cm) − 6.755 × age (years) for men and 655 + 9.563 × weight (kg) + 1.850 × height (cm) − 4.676 × age (years) for women. We defined patients with HF above BEE as the high-energy group and patients with HF below BEE as the low-energy group.

### 2.4. Malnutrition Screening Tools

Both the GNRI score [6] and the CONUT score [7] are nutrition screening tools. The GNRI was calculated using the following formula: 1.489 × serum albumin (g/L) + 41.7 × (body weight in kilograms/ideal body weight). The ideal body weight was calculated using the following formula: 22 × square of height in meters. A score > 98 is considered normal; scores of 92 to 98, 82 to 91, and <82 reflect mild, moderate, and severe malnutrition, respectively. The CONUT score takes into account serum albumin levels, cholesterol levels, and total lymphocyte counts. A score of 0 to 1 is considered normal, while scores of 2 to 4, 5 to 8, and 9 to 12 reflect mild, moderate, and severe malnutrition, respectively.

### 2.5. MAGGIC Risk Score

The MAGGIC risk score is a well-validated risk score for predicting the 1-year mortality of outpatients with HF [5]. It is calculated for each patient based on 13 variables (age, sex, current smoker, systolic blood pressure, body mass index, serum creatinine, diabetes, chronic obstructive pulmonary disease, New York Heart Association functional class, time since diagnosis of HF, LVEF, nonprescription of beta-blockers, and nonprescription of angiotensin-converting enzyme inhibitors or angiotensin receptor blockers).

### 2.6. Outcomes and Follow-Up

Patients were followed until 31 March 2020. The median follow-up period in this study was 19 (13–24) months. The primary outcome was the composite outcome of all-cause death or readmission due to worsening HF. Decisions regarding the need for readmission due to HF were made according to the directions of the treating physicians and according to standard practice. The exploratory secondary outcomes comprised each outcome taken separately.

### 2.7. Statistical Analyses

Statistical analysis was performed using JMP Pro version 16 (SAS Institute, Cary, NC, USA) and R version 4.1.3 (R Foundation for Statistical Computing, Vienna, Austria). Categorical variables are presented as numbers (%) and were compared using the χ^2^ test. Continuous variables are presented as the mean ± SD or median [25th percentile, 75th percentile] and were compared using Student’s *t*-test. The Wilcoxon test was applied for nonparametric comparisons. To identify determinants of low energy intake, we selected variables showing *p* < 0.05 in the univariate analysis and performed multivariable logistic regression analysis. OR indicates the unit odds ratio, and HR indicates the unit hazard ratio. The *p* value was calculated with a Wald test. The log-rank test was used for event-free survival curves. In all analyses, statistical significance was defined by the criterion of *p* < 0.05.

## 3. Results

### 3.1. General Observations

Based on the BEE calculation, 38 patients with HF were classified into the low-energy group, and 101 patients with HF were classified into the high-energy group. The patient characteristics are summarized in Table 1. Compared to the patients in the high-energy group, the patients in the low-energy group were predominantly female (low-energy group vs. high-energy group, 92% vs. 24%, *p* < 0.0001), were less likely to smoke (current and past smoker) (current/past/never, 5%/16%/79% vs. 10%/42%/49%, *p* = 0.005), had a lower rate of ischemic heart diseases (8% vs. 33%, *p* = 0.002) and had a higher LVEF (44.6 ± 16.4% vs. 38.3 ± 15.6%, *p* = 0.041). Vital, physical, laboratory, echocardiographic, and nutritional data at both admission and at discharge are shown in Table 1. At admission, although patients in the low-energy group showed a higher heart rate (102.5 ± 29.1/min vs. 90.9 ± 23.8/min, *p* = 0.017), there were no significant differences in other parameters, including BNP (651.7 [interquartile range (IQR): 447.0, 893.8] pg/mL vs. 756.0 [IQR: 504.9, 1203.4] pg/mL, *p* = 0.525) or nutritional items, such as the GNRI (*p* = 0.572) and CONUT (*p* = 0.737). At discharge, there was no significant difference in the BNP (261.0 [IQR: 124.7, 460.0] pg/mL vs. 259.7 [138.0, 520.0] pg/mL, *p* = 0.677) or MAGGIC risk score (*p* = 0.139), which is a prognostic index. Furthermore, no significant differences in the qualitative nutritional status ratios or CONUT and GNRI scores were noted between the two groups (Table 2). There was no significant difference in medications at discharge (Table 3) between the two groups.

### 3.2. Multivariate Analysis of the Determinants of Low Energy Intake

The results of the multivariate analysis are summarized in Table 4. Being female (*p* < 0.001) was an independent predictor of low energy intake.

### 3.3. Prognosis of Patients with Low Energy Intake

A total of 26 fatal cases, including 21 cardiovascular disease (CV) deaths and 5 non-CV deaths, were observed. Among the fatal CV cases, 19 were due to HF, and 2 were due to stroke. Among the non-CV fatal cases, two were due to cancer, one to senility, one was a postoperative complication of gastroenterological disease, and one case had an unknown cause. In the low-energy group, 10 out of 11 deaths (90%) were CV deaths (9 HF and 1 stroke), while 11 out of 15 deaths (73%) in the high-energy group were CV deaths (10 HF and 1 stroke), indicating a similar ratio of CV deaths between the two groups (*p* = 0.356).

Rehospitalization for worsening HF was observed in 11 (29%) patients in the low-energy group and 39 (39%) patients in the high-energy group, with similar levels of rehospitalization for worsening HF between the two groups (*p* = 0.290).

**Figure 2:**
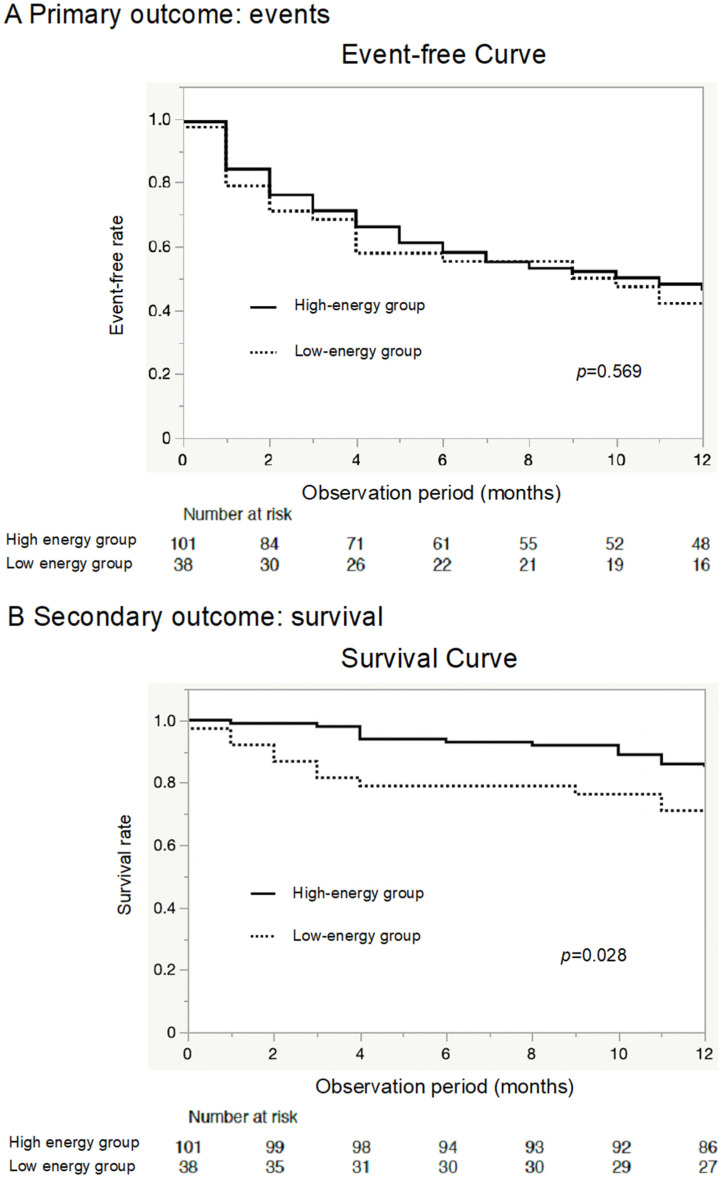

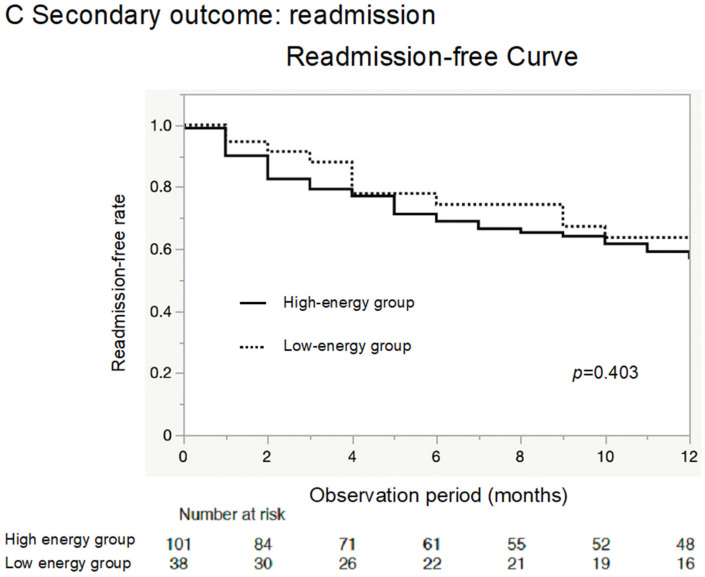
Primary outcome, all-cause mortality, and readmission due to worsening heart failure. The Kaplan–Meier (KM) curve shows that the rate of the primary outcome (all-cause mortality or heart failure readmission) was similar between the two groups (**A**). The KM curve shows that elderly heart failure patients in the low-energy group were associated with relatively poor outcomes for mortality compared to the high-energy group (**B**). However, the rehospitalization rates for heart failure were similar between the two groups (**C**).

The Kaplan–Meier (KM) curve of the primary outcome showed that the two groups had equivalent event rates (*p* = 0.569) (Figure 2A). On the other hand, the KM curve of all-cause death demonstrated a worse prognosis in the low-energy group than in the high-energy group (*p* = 0.028, Figure 2B), although the two groups showed equivalent readmission rates (*p* = 0.403, Figure 2C).

## 4. Discussion

There were three major findings of this study. First, there was no significant difference in the BNP levels between patients with adequate and inadequate energy intake, as classified according to the energy requirements determined using the Harris–Benedict equation, at admission and at discharge. Second, the nutritional scores and the prognostic index for patients with HF were similar between the two groups. Third, the low-energy group showed similar rates of the primary endpoint (composite outcome of all-cause death or readmission due to worsening HF) to the high-energy group.

Poor nutritional status in patients with HF is generally recognized to increase the risk of future mortality. In particular, the GNRI is thought to have the greatest incremental value in predicting the risk of death among HF patients [16]. The GNRI is associated with the duration of hospital stay, congestion, cardiovascular events, and long-term prognosis among patients with HF [17]. However, in this study, regardless of the similar GNRI scores and status between the two groups, the low-energy group showed worse outcomes. The CONUT score is a nutritional index derived from albumin levels, total cholesterol levels, and lymphocyte counts [7]. On the other hand, albumin levels are influenced by diuretics, and total cholesterol levels are influenced by statins. Therefore, it may not be appropriate to use the CONUT score as a nutritional indicator in HF patients who frequently use these drugs. Because of these factors, the CONUT scores probably did not differ significantly between the two groups. Various studies have reported that patients with HF and high BNP levels have a poor prognosis [18,19,20]. Many studies on HF related to poor nutrition have reported significantly lower BNP levels in malnutrition groups, with a poor prognosis [5,14,21,22,23]. BNP is a biochemical marker that sensitively reflects the degree of ventricular overload, so it is possible that the undernourished groups in these studies may have had worse control of HF than the control groups. However, we found no significant differences in BNP levels between the groups. Furthermore, the medical treatment was similar between the groups. Patients with HF and malnutrition are generally associated with a low body mass index (BMI). Patients with HF and a low BMI have a poor prognosis [24,25,26] because cases of low BMI include those with cardiac cachexia caused by chronic inflammation or physiologic abnormalities, and cardiac cachexia has a serious negative impact on HF [27]. The patients in this study had appropriate BMIs, and it was thought that there would be no prognostic impact of an abnormal BMI. In the present study, the proportion of women was higher in the low-energy group than in the high-energy group, while women generally tended to have a lower risk of mortality. If the sex ratio is equal, there might be a significant difference in events, especially mortality. From the above results, this study showed that a low energy intake is associated with higher future rates of mortality, even in the context of similar BNP levels, body weights, and nutritional status scores.

Energy deprivation induces alterations in intestinal permeability and digestive ability [28,29,30]. Genton et al. demonstrated that the gut microbiota’s composition and function were altered in nutritionally depleted states [29]. An altered microbial composition, which is caused by the irreversible loss of gut microbes, is referred to as dysbiosis [31] and is considered to play a role in cardiovascular diseases [32]. Indeed, patients with HF in the low-energy group may have experienced dysbiosis. Additionally, energy deprivation influences cytokine levels (e.g., increased serum levels of tumor necrosis factor-alpha (TNF-a) and decreased levels of interleukin-2 [33]) and immune-related cells (e.g., diminished phagocytic activity of macrophages [34] and decreased natural killer cell activity and T-cell proliferation [35]). However, whether these conditions are related to a deficient intake of energy, along with whether they are aggravated by components other than nutritional intake, such as chronic inflammation, remains unclear.

To the best of our knowledge, no previous studies have evaluated the association between energy intake and the mid-term prognosis of elderly patients with HF. Ill patients, including those with HF, often have energy needs that are greater than expected based on their body composition [36]. The Harris–Benedict equation (HBE) [10] is a classical predictor of basal energy expenditure [11]. Although the HBE is thought to underestimate energy requirements in elderly patients [12], the HBE may have just the right balance for assessing the energy requirements of elderly patients with HF.

Several previous observational surveys have shown that insufficient food intake is associated with adverse outcomes. Yoshida et al. found that inadequate food intake in relatively young patients with acute cardiac insufficiency increased mortality and HF rehospitalization rates. Hersberger et al. demonstrated that nutritional support for patients with HF reduced the risk of short-term (30-day) mortality [37]. The results of these studies support the conclusion that inadequate energy intake at discharge is associated with a poor prognosis and are in agreement with our findings among patients with HF.

There were no significant differences in readmission rates due to worsening HF between the two groups (the low-energy group and the high-energy group). The background of the patients enrolled in this study was approximately the same. Although there were no significant differences in age or NYHA classification/BNP/Hb levels/renal function at discharge between the two groups, both groups were very old, had low LVEF and poor renal function, and were hospitalized approximately two times on average for HF exacerbations. The patients in this study were considered to have late stage C to stage D HF and were more likely to have acute exacerbations of chronic HF due to factors other than energy intake, which would have resulted in a higher rehospitalization rate [38]. Therefore, we consider that the rehospitalization rate was not significantly different between the two groups.

This study has several limitations. It was an observational study, so some unmeasured variables might have influenced the outcomes. Additionally, it had a relatively small sample size and was conducted at a single center. The medications in this study did not include some new types of drugs, such as sodium glucose cotransporter 2 inhibitors (SGLT2i) or angiotensin receptor–neprilysin inhibitors (ARNi). This study is based on patient data obtained from 2018, so not all patients were taking these new types of drugs, and their impact does not need to be considered. Finally, we investigated energy intake only in the last three days before discharge, and changes in energy intake during hospitalization may have different implications.

## 5. Conclusions

In elderly patients with HF, the composite endpoint (all-cause mortality within one year and rehospitalization for worsening HF) did not differ significantly between the two groups diagnosed using the Harris–Benedict formula with regard to energy intake. On the other hand, inadequate energy intake diagnosed with the formula was associated with a higher incidence of all-cause mortality within one year in elderly HF patients. The monitoring of energy intake may therefore be useful in predicting mortality risk in elderly patients with HF, and the basal energy requirement derived from the Harris–Benedict equation may serve as an indicator for such monitoring. However, in the present study, no intervention using the Harris–Benedict formula for basal energy expenditure as a reference was performed, and further studies with interventions for energy intake are needed to verify whether it is a useful indicator for elderly patients with HF.

## Figures and Tables

**Table 1:** Baseline clinical characteristics and clinical data at both admission and discharge.

	Low-Energy Group	High-Energy Group	*p* Value
	(*n* = 38)	(*n* = 101)	
Baseline Clinical Characteristics			
Age, years	81.2 ± 13.7	79.1 ± 10.3	0.336
Female	35 (92)	24 (24)	<0.0001
Hospital stay, days	22 [17, 30]	20 [15, 30]	0.755
Number of previous hospitalizations	1.8 ± 1.8	2.0 ± 2.2	0.472
Family living together	24 (63)	71 (70)	0.422
Hypertension	29 (76)	75 (74)	1.000
Diabetes mellitus	14 (37)	36 (36)	0.333
Dyslipidemia	13 (34)	39 (39)	0.697
Smoking (current/past/never)	2/6/30	10/42/49	0.005
Atrial fibrillation	18 (47)	42 (42)	0.569
Ischemic heart disease	3 (8)	33 (33)	0.002
Left ventricular ejection fraction, %	44.6 ± 16.4	38.3 ± 15.6	0.041
EF category (pEF/mrEF/rEF)	18/3/17	30/17/56	0.097
Vital, physical, laboratory, echocardiological, and nutritional data at admission			
Body weight, kg	54.5 ± 13.3	57.2 ± 11.9	0.306
Heart rate, beats/min	102.5 ± 29.1	90.9 ± 23.8	0.017
Systolic blood pressure, mmHg	140.4 ± 30.9	133.4 ± 25.8	0.180
Diastolic blood pressure, mmHg	76.2 ± 19.8	76.3 ± 18.3	0.987
Body mass index, kg/m^2^	23.2 ± 3.9	23.0 ± 3.9	0.865
NYHA classification (1/2/3/4)	0/5/22/11	0/31/54/18	0.080
Hemoglobin, g/dL	11.4 ± 2.0	11.9 ± 1.9	0.232
Creatinine, mg/dL	1.1 ± 0.5	1.4 ± 0.8	0.111
BUN, mg/dL	27.0 ± 13.2	26.3 ± 14.5	0.809
eGFR, mL/min/1.73 m^2^	40.0 [28.2, 54.8]	47.6 [32.6, 59.1]	0.247
BNP (pg/mL)	651.7 [447.0, 893.8]	756.0 [504.9, 1203.4]	0.525
CRP (mg/dL)	0.8 ± 1.4	1.1 ± 2.1	0.446
Troponin I (pg/mL)	51.1 [21.0, 230.0]	109.8 [35.9, 465.9]	0.216
Albumin (mg/dL)	3.6 ± 0.4	3.6 ± 0.4	0.886
GNRI	99.3 ± 9,8	98.0 ± 9.8	0.572
CONUT	2.0 ± 1.7	2.1 ± 1.7	0.737
Vital, physical, laboratory, echocardiological, and nutritional data at discharge			
Body weight, kg	48.6 ± 13.1	52.8 ± 10.5	0.056
Heart rate, beats/min	74.4 ± 14.3	72.3 ± 11.1	0.354
Systolic blood pressure, mmHg	119.4 ± 16.7	113.6 ± 15.8	0.060
Diastolic blood pressure, mmHg	63.9 ± 10.1	62.6 ± 10.6	0.513
Body mass index, kg/m^2^	21.0 ± 4.3	21.1 ± 3.5	0.862
NYHA classification (1/2/3/4)	6/28/4/0	33/65/3/0	0.101
Hemoglobin, g/dL	12.0 ± 2.0	12.0 ± 1.8	0.899
Creatinine, mg/dL	1.2 ± 0.5	1.5 ± 1.1	0.118
BUN, mg/dL	31.2 ± 18.7	31.4 ± 19.7	0.951
eGFR, mL/min/1.73m^2^	40.1 [28.4, 52.2]	43.2 [34.6, 52.9]	0.502
BNP, pg/mL	261.0 [124.7, 460.0]	259.7 [138.0, 520.0]	0.677
Albumin, mg/dl	3.6 ± 0.3	3.6 ± 0.4	0.835
GNRI	91.8 ± 11.4	93.1 ± 7.6	0.548
CONUT	1.3 ± 1.5	1.2 ± 1.4	0.793
MAGGIC risk score	24.8 ± 6.4	26.7 ± 6.6	0.139

Values are presented as *n* (%), median [25th percentile, 75th percentile] or mean ± SD. Abbreviations: EF, ejection fraction; pEF, preserved ejection fraction; mrEF, mildly reduced ejection fraction; rEF, reduced ejection fraction, NYHA, New York Heart Association; BUN, blood urea nitrogen; eGFR, estimated glomerular filtration rate; BNP, brain natriuretic peptide; CRP, C-reactive protein; GNRI, Geriatric Nutritional Risk Index; CONUT, Controlling Nutritional Status; MAGGIC, Meta-Analysis Global Group in Chronic Heart Failure.

**Table 2:** Nutritional assessment data.

	Low-Energy Group	High-Energy Group	*p* Value
	(*n* = 38)	(*n* = 101)	
At admission			
GNRI (normal/mild/moderate/severe)	27/5/4/2	63/16/22/0	0.054
CONUT (normal/mild/moderate/severe)	15/20/3/0	41/48/12/0	0.755
At discharge			
GNRI (normal/mild/moderate/severe)	10/11/12/5	26/32/38/3	0.402
CONUT (normal/mild/moderate/severe)	25/0/13/0	61/38/2/0	0.616

Values are presented as *n*. Abbreviations: GNRI, Geriatric Nutritional Risk Index; CONUT, Controlling Nutritional Status.

**Table 3:** Medication at discharge.

	Low-Energy Group	High-Energy Group	*p* Value
	(*n* = 38)	(*n* = 101)	
ACEi/ARB	21 (55)	67 (66)	0.242
β blocker	24 (68)	67 (66)	0.816
MRA	7 (18)	36 (36)	0.0502
Loop diuretic	33 (87)	92 (91)	0.529
Tolvaptan	12 (32)	32 (32)	1.000
Calcium blocker	10 (26)	30 (30)	0.834
Digitalis	2 (5)	1 (1)	0.181
Oral inotropic drug	2 (5)	12 (12)	0.350
Statin	11 (29)	32 (32)	0.839

Values are presented as number (%). Abbreviations: ACEi, angiotensin-converting enzyme inhibitor; ARB, angiotensin II receptor blocker; MRA, mineralocorticoid receptor antagonist.

**Table 4:** Multivariate analysis: determinants of low energy intake.

	Odds Ratio	95% CI	*p* Value
Female	82.760	8.967–872.729	<0.001
Smoking habit (y/*n*)	4.937	0.505–41.945	0.092
Ischemic heart disease	0.439	0.095–2.115	0.283
HR at admission	1.017	0.996–1.038	0.097
sBP at discharge	1.022	0.989–1.053	0.172
MRA at discharge	0.797	0.225–2.871	0.725
LVEF	1.389	0.973–1.065	0.755

Abbreviations: BP, blood pressure; CI, confidence interval, HR: heart rate; LVEF, left ventricular ejection fraction; MRA, mineralocorticoid receptor antagonist.

## Data Availability

The data presented in this study are available upon request from the corresponding author. The data are not publicly available due to privacy and ethical restrictions.
